# Alpha1-antitrypsin improves survival in murine abdominal sepsis model by decreasing inflammation and sequestration of free heme

**DOI:** 10.3389/fimmu.2024.1368040

**Published:** 2024-03-18

**Authors:** Jan D. Zemtsovski, Srinu Tumpara, Sonja Schmidt, Vijith Vijayan, Andreas Klos, Robert Laudeley, Julia Held, Stephan Immenschuh, Florian M. Wurm, Tobias Welte, Hermann Haller, Sabina Janciauskiene, Nelli Shushakova

**Affiliations:** ^1^ Department of Nephrology and Hypertension, Hannover Medical School, Hannover, Germany; ^2^ Department of Respiratory Medicine, Member of the German Center for Lung Research (DZL), Biomedical Research in Endstage and Obstructive Lung Disease Hannover (BREATH), Hannover Medical School, Hannover, Germany; ^3^ Phenos GmbH, Hannover, Germany; ^4^ Institute for Transfusion Medicine and Transplant Engineering, Hannover Medical School, Hannover, Germany; ^5^ Institute of Medical Microbiology and Hospital Epidemiology, Hannover Medical School, Hannover, Germany; ^6^ Faculty of Life Sciences, École Polytechnique Fédérale de Lausanne, Lausanne, Switzerland

**Keywords:** mice, sepsis, alpha1-antitrypsin, free heme, inflammation, cytokines, neutrophils, macrophages

## Abstract

**Background:**

Excessive inflammation, hemolysis, and accumulation of labile heme play an essential role in the pathophysiology of multi-organ dysfunction syndrome (MODS) in sepsis. Alpha1-antitrypsin (AAT), an acute phase protein with heme binding capacity, is one of the essential modulators of host responses to inflammation. In this study, we evaluate the putative protective effect of AAT against MODS and mortality in a mouse model of polymicrobial abdominal sepsis.

**Methods:**

Polymicrobial abdominal sepsis was induced in C57BL/6N mice by cecal ligation and puncture (CLP). Immediately after CLP surgery, mice were treated intraperitoneally with three different forms of human AAT—plasma-derived native (nAAT), oxidized nAAT (oxAAT), or recombinant AAT (recAAT)—or were injected with vehicle. Sham-operated mice served as controls. Mouse survival, bacterial load, kidney and liver function, immune cell profiles, cytokines/chemokines, and free (labile) heme levels were assessed. In parallel, *in vitro* experiments were carried out with resident peritoneal macrophages (MPMΦ) and mouse peritoneal mesothelial cells (MPMC).

**Results:**

All AAT preparations used reduced mortality in septic mice. Treatment with AAT significantly reduced plasma lactate dehydrogenase and s-creatinine levels, vascular leakage, and systemic inflammation. Specifically, AAT reduced intraperitoneal accumulation of free heme, production of cytokines/chemokines, and neutrophil infiltration into the peritoneal cavity compared to septic mice not treated with AAT. *In vitro* experiments performed using MPMC and primary MPMΦ confirmed that AAT not only significantly decreases lipopolysaccharide (LPS)-induced pro-inflammatory cell activation but also prevents the enhancement of cellular responses to LPS by free heme. In addition, AAT inhibits cell death caused by free heme *in vitro*.

**Conclusion:**

Data from the septic CLP mouse model suggest that intraperitoneal AAT treatment alone is sufficient to improve sepsis-associated organ dysfunctions, preserve endothelial barrier function, and reduce mortality, likely by preventing hyper-inflammatory responses and by neutralizing free heme.

## 1 Introduction

Sepsis and septic shock remain high-risk factors for mortality. According to the World Health Organization’s report, in 2017, there were 48.9 million cases and 11 million sepsis-related deaths worldwide (1). In particular, mortality in patients with septic shock remains high because there are limited treatment options other than antibiotics (2). Important points in the treatment of acute sepsis are early diagnosis and targeted treatment in the first few hours.

Activation of the innate immune system is the initial host defense against invading microorganisms, which is important for the induction of an adaptive immune response and pro-/anti-inflammatory mediators to prevent organ damage. The coagulation, fibrinolysis, and complement systems as well as endothelial dysfunction play a role (3–5), and hyper-inflammation in this scenario sometimes does more harm than good. Therefore, understanding the role of immune cells and released inflammatory molecules during acute sepsis is essential to develop better therapeutic tools (6, 7).

Sepsis is characterized by extensive death of hematopoietic and parenchymal cells and subsequent systemic release of cell-free hemoglobin and cell-free heme. Free heme has been shown to play a central role in the pathogenesis of severe sepsis (8, 9). During acute sepsis, there is also a strong shift toward proteolysis, and therefore, acute phase proteins (APPs) with anti-protease activities are of particular interest. Human alpha1-antitrypsin (AAT) is an archetypal member of the serine protease inhibitors and one of the most important acute phase proteins with broad immunomodulatory functions. Under health conditions, AAT plasma levels in humans are between 1 and 2 g/L and increase a few times over the normal range in acute inflammation or infection (10). It is important to point out that AAT not only interacts with target proteases and inhibits their activity but also binds and neutralizes various inflammatory substances such as free radicals, chemokines [interleukin 8 (CXCL8) and leukotriene B4 (LTB4)], cytokines [tumor necrosis factor (TNF)], and complement factors. Like albumin, AAT also interacts with free heme and neutralizes its toxicity (10, 11). The fact that the half-life of circulating AAT is prolonged during bacteremia suggests that AAT is an important protection against organ damage (12). Consistent with the latter, a previous study showed that AAT reduces bacterial burden in the rodent sepsis model *in vivo* (13). In contrast, administration of high-dose recombinant AAT *Pittsburgh* (dysfunctional variant) in a primate sepsis model showed exacerbation of septic shock mainly due to high levels of cleaved AAT, which elicited a strong immune response (14).

During acute sepsis, AAT levels can be severely reduced due to inhibition of activated target proteases as well as high non-specific cleavage, mainly by cysteine and metalloproteases. In support of this notion, recent studies have highlighted the putative value of cleaved AAT fragments as biomarkers of sepsis severity (15, 16). Therefore, timely augmentation with exogenous AAT may be beneficial to compensate for the loss of endogenous AAT protein (17). Today, there are few commercial preparations of AAT purified from human plasma that are used to treat patients with congenital AAT deficiency, and these preparations are also being tested for their therapeutic potential outside of AAT deficiency (18). There are also recombinant forms of AAT, which are developed to be used as therapeutics instead or in parallel to plasma-purified AAT (19, 20).

In this study, we used a cecal ligation and puncture (CLP) mouse model to investigate plasma-derived native (nAAT), oxidized nAAT (oxAAT) lacking anti-elastase activity, and inhibitory active recombinant AAT (recAAT) expressed in Chinese hamster ovary (CHO) cells for their effects on factors associated with acute sepsis. We assessed survival, bacterial load, and biomarkers associated with inflammation and organ dysfunction. We sought to obtain experimental data on whether the use of AAT as an early treatment of acute sepsis may have a putative benefit that can be further investigated in the clinical setting.

## 2 Materials and methods

### 2.1 Alpha1-antitrypsin proteins

Plasma-purified human AAT (99% purity, Zemaira, CSL Behring, Kankakee, IL, USA) was used for experiments after buffer exchange to the sterile Hank’s Balanced Salt Solution (HBSS) (Merck Millipore, Darmstadt, Germany) using 10K centrifugal filter columns (Sartorius, Göttingen, Germany). The protein concentration was determined using the BCA Protein Assay Kit (Pierce™, Rockford, IL, USA) according to the instructions of the supplier. The oxAAT was prepared from AAT (Zemaira®) by adding *N*-chlorosuccinimide (Sigma-Aldrich, Merck, Darmstadt, Germany) at a molar ratio of 1:20 (AAT: *N*-chlorosuccinimide) for 20 min at room temperature. Afterward, to remove the *N*-chlorosuccinimide, AAT preparations were washed with phosphate-buffered saline (PBS; Sigma-Aldrich, St. Louis, MO, USA) using Vivaspin 20 centrifugal filter devices with a cutoff of 10K. The oxAAT did not form complexes with elastase and showed a retarded electrophoretic mobility relative to a native AAT. A highly purified (90%), glycosylated form of recombinant AAT protein produced in CHO cells was a gift from ExcellGene, Monthey, Switzerland.

### 2.2 Mice

Ten- to twelve-week-old male C57BL/6N mice (20 to 25 g) were obtained from Charles River Laboratories (Sulzfeld, Germany). Mice were maintained on mouse chow and tap water *ad libitum* in a temperature-controlled chamber at 24°C with a 12:12-h light–dark cycle. All procedures were approved by the local committee for the care and use of laboratory animals (Lower Saxony Office for Consumer Protection and Food Safety, LAVES no. 21-3761) and were performed in accordance with international guidelines on animal experimentation.

### 2.3 Cecal ligation and puncture model of polymicrobial sepsis

Polymicrobial sepsis in mice was induced by CLP surgery. In brief, mice were anesthetized with isoflurane (induction of 3%, maintenance of 1.5%, and oxygen flow of 3 L/min), and a 1-cm ventral midline abdominal incision was made. The cecum was then exposed, ligated with 4-0 silk sutures, and punctured through using a 24-gauge needle. The punctured cecum was gently squeezed to expel a 1- to 2-mm droplet of fecal material and returned to the abdominal cavity. The incision was closed in layers using 4-0 surgical sutures. Mice were fluid-resuscitated with pre-warmed normal saline (500 μL) intraperitoneally (i.p.) immediately after the procedure. Sham animals underwent the same procedure except for CLP. All experiments were performed at the same time of day. For pre-operative treatment, 0.1 mg/kg buprenorphine and 100 mg/kg metamizole were administered s.c. For post-operative analgesia, animals were s.c. injected with 0.1 mg/kg buprenorphine twice daily for 3 days.

### 2.4 Survival analysis

For survival analysis, mid-grade sepsis was induced by CLP surgery with ligation of 50% of cecum length (21). Mice were treated i.p. with nAAT, oxAAT, or recAAT (200 mg/kg body weight) or HBSS (vehicle treatment) immediately after CLP or sham surgery. The survival was monitored up to 14 days after surgery. Blood samples were collected 3 days prior to and at 24 h after surgery under light isoflurane anesthesia. EDTA plasma samples were generated, stored at −80°C, and used for the quantification of TNF, interleukin 6 (IL-6), and chemokine ligand 2 (CCL2).

### 2.5 Short-term high-grade sepsis and assays

Short-term high-grade sepsis was induced by CLP surgery with ligation of 75% of cecum length (21), and immediately after surgery, mice were treated i.p. with recAAT or vehicle (HBSS buffer). Twenty hours after surgery, mice were anesthetized with isoflurane and, after blood collection, were sacrificed. EDTA plasma samples were generated, and peritoneal lavage (PL) was performed using 3 mL of PBS and collected in tubes without anti-coagulant. The volume of collected PL fluid was measured in each sample, and the total cell number was determined using a hemocytometer (Neubauer Zählkammer, Gehrden, Germany). Plasma and PL fluids were stored at −80°C for further analyses.

### 2.6 Microvasculature permeability assay

In separate experiments, the Evans blue assay was performed to estimate microvascular permeability. Immediately after CLP surgery, 200 µL of 0.25% wt/vol Evans blue dye (Sigma-Aldrich) in PBS was injected intravenously. Blood sampling was performed under isoflurane anesthesia at 20 h after CLP surgery, the mice were sacrificed, and PL was performed. The concentration of Evans blue dye in appropriate dilutions of plasma and PL fluid samples was measured spectrophotometrically at 620 nm. The following formula was used to correct the optical densities for contamination with heme pigments: E620 (corrected) = E620 (raw) − (E405 (raw) × 0.014. Plasma exudation was quantified as the ratio of extinction in PL fluid to extinction in plasma.

### 2.7 Cytokine detection in plasma and PL fluid

Levels of the pro-inflammatory cytokines TNF, IL-6, and CCL2 were quantified in plasma and PL fluid by bead-based flow cytometry assay (CBA Kit; BD Biosciences, Heidelberg, Germany) in accordance with the instructions of the manufacturer.

### 2.8 FACS analysis of cell populations in PL fluid

The inflammatory cell populations in the PL fluid were analyzed by flow cytometry using a fluorescence-activated cell sorting (FACS) Canto cytometer (BD Biosciences, Franklin Lakes, NJ, USA). The following commercial monoclonal antibodies (BioLegend, San Diego, CA, USA) were used for the detection of leukocytes, macrophages, polymorphonuclear neutrophils (PMNs), B cells, and T cells, respectively: anti-CD11b, anti-F4/80, anti-Gr1, anti-CD115, anti-TCR β, and anti-CD19. Further analyses were performed using FlowJo software (Tree Star, Ashland, OR, USA).

### 2.9 Semi-quantification of AAT levels in plasma and PL fluid

Levels of endogenous AAT were estimated in plasma and PL fluid samples obtained at 20 h post-surgery from untreated septic and sham mice by Western blotting. In brief, 1 µL of plasma samples and 5 µL of PL fluid samples were separated on 10% sodium dodecyl sulfate–polyacrylamide gels (SDS–PAGE). From gels, proteins were transferred onto polyvinylidene fluoride membrane by semidry Western blotting. For specific detection of AAT, primary rabbit polyclonal anti-AAT antibody was used at a dilution of 1:800 (DAKO, Glostrup, Denmark). The immune complexes were visualized using appropriate secondary horseradish peroxidase-conjugated antibodies (DAKO, Denmark) at a dilution of 1:10,000 and enhanced chemiluminescence (ECL) Western blotting substrate (Thermo Fisher Scientific, Grand Island, NY, USA). The density of the specific bands was quantified using Image Lab v5.2.1 software (Bio-Rad, Hercules, CA, USA).

### 2.10 Labile heme quantification in PL fluid using Apo-horseradish peroxidase assay

Measurement of labile heme concentration in PL fluid samples was performed in 96-well plates as previously described (22). Briefly, 5 µL of an appropriately diluted PL fluid sample was added to 95 µL of HBSS buffer containing 0.75 µM Apo-Horseradish Peroxidase (Apo-HRP) (BBI Solutions, Gwent, UK) and incubated for 10 min at 4°C. Simultaneously, hemin standards ranging from 0.25 to 2.5 nM were prepared in HBSS buffer in a final volume of 100 μL from a stock solution of 25 nM hemin (Frontier Scientific, Logan, UT, USA) and incubated for 10 min at 4°C. Then, 5 μL of each sample and standard were added to a 96-well plate. To start the assay, 200 μL of 3,3′,5,5′-tetramethylbenzidine (TMB) substrate was added per well. To determine the concentration of labile heme, the absorbance was measured at 652 nm for 2–3 min. The time point at which the highest hemin standard induced an absorbance from 1.6 to 2 was used.

### 2.11 Bacteria burden in CLP mice in peritoneal lavage fluid, spleen, and blood

EDTA blood, PL fluid, and spleens were obtained from septic mice treated with vehicle, nAAT, or oxAAT at 20 h after induction of high-grade sepsis. The peritoneal lavage fluid was serially diluted 1:10 in PBS five times (4°C). The spleen was submerged for 1 min in 70% ethanol to remove potential contaminations of its surface before rinsing the organ briefly with PBS. Then, the organ was homogenized using 100-µm BD Falcon™ Cell Strainer and pistil, thereby rinsing the sieve with 2 mL of sterile PBS. The spleen homogenate at a volume of 500 µL was diluted 1:10 in PBS. Then, 100 µL of the undiluted lavage fluid, the spleen homogenate, and each dilution was transferred onto agar plates for culture at 35°C. Columbia agar containing 5% sheep blood plates (Becton Dickinson, Franklin Lakes, NJ, USA; 4354071) used for the growth of Gram-positive and Gram-negative bacteria and MacConkey agar plates (Mast Diagnostica GmbH, Reinfeld/Stormarn, Germany; 202010) used for growth of Gram-negative bacteria were incubated under aerobic conditions; Schaedler agar plates (Becton Dickinson, 4354084) incubated under anaerobic conditions in an air-tight plastic container (AnaeroGen 3.5L, Thermo Fisher Scientific) were used to permit growth of Gram-positive and Gram-negative anaerobic bacteria. EDTA blood was diluted 1:10 in PBS, and 100 µL of the undiluted and diluted samples was evenly spread on a Columbia agar and Schaedler agar for culture under aerobic or anaerobic conditions, respectively. In all cases, after approx. 48 h of culture, bacterial colonies on the plates were counted. The number of colonies forming units in the original sample was calculated considering the applied volume and dilution factor.

### 2.12 Experiments with murine cell cultures and analyses

Immortalized mouse peritoneal mesothelial cells (MPMC) were cultivated as described previously (23). Briefly, the cells were grown on 6-well or 24-well cell culture plates to 80% confluence in Roswell Park Memorial Institute (RPMI) medium GlutaMAX™ (Thermo Fisher Scientific, Grand Island, NY, USA) supplemented with 10% fetal bovine serum (FBS), 1% Penicillin/Streptomycin (Sigma-Aldrich, St. Louis, MO, USA), 0.4 mg/mL hydrocortisone (Sigma-Aldrich, St. Louis, MO, USA), 1% Insulin-Transferrin-Selenium sodium pyruvate (Thermo Fisher Scientific, Waltham, MA, USA), and 10 U/mL recombinant mouse interferon γ (IFN-γ; Cell Sciences, Canton, MA, USA) at 33°C (permissive conditions). The cells were differentiated for 3 days in the same medium at 37°C without IFN-γ (non-permissive conditions) and starved overnight in a serum-free medium. MPMC were stimulated for 24 h with different concentrations of lipopolysaccharide (LPS) (E. coli O111:B4, Sigma-Aldrich), free hemin (Sigma-Aldrich), or recAAT separately or in combination. The production of pro- and anti-inflammatory cytokines TNF, IL-6, IL-10, and CCL2 were measured in the conditioned medium as described above for PL fluid samples.

Resident peritoneal macrophages (MPMΦ) were obtained from healthy C57BL/6N mice by peritoneal lavage (2 × 5 mL PBS). After centrifugation at 300 g for 10 min at 4°C, the supernatants were decanted, and the remaining pellets were washed with RPMI GlutaMAX™ medium supplemented with 10% FBS, resuspended in the same medium, and distributed into 24-well culture plates at a concentration of 1.0 × 10^6^ cells/mL per well. Plates were incubated overnight at 37°C, 5% CO_2_, and 95% humidity to allow macrophage adhesion.

Non-adherent cells were removed by vigorous washing with RPMI 1640 medium. MPMΦ were starved for 4 h in 1% FBS/RPMI 1640 medium and then stimulated with 5 ng/mL LPS, with increasing concentrations of recAAT (0.1, 1, 10, and 100 µg/mL) or with LPS/recAAT combinations for 24 h. The MPMΦ medium without stimuli served as a control. The release of pro- and anti-inflammatory cytokines, TNF, IL-6, IL-10, and CCL2, was examined in the conditioned medium as described above for PL fluid samples.

### 2.13 RNA isolation and real-time polymerase chain reaction

RNA was isolated using NucleoSpin RNA II Kit (Macherey-Nagel GmbH & Co. KG, Duren, Germany) and reversely transcribed with M-MLV-RT (Promega GmbH, Mannheim, Germany) according to the manufacturer’s instructions. Real-time polymerase chain reactions were performed in triplicates on a LightCycler 480 or LightCycler 96 using SYBR Green (Roche, Grenzach-Wyhlen, Germany). Primer sequences were selected using PrimerBank and are reported in the **Supplementary Methods**. HPRT1 was used as a reference gene. Primers for HPRT1 were obtained from Biomol GmbH (Hamburg, Germany).

### 2.14 Apoptosis assay

Apoptosis was assessed using fluorescein isothiocyanate (FITC) Annexin V Apoptosis Detection Kit with Propidium Iodide solution (PI) (BioLegend). To distinguish early-stage apoptotic cells from late-stage apoptotic and necrotic cells, Annexin V and Propidium Iodide solution PI were used according to the manufacturer’s instructions.

### 2.15 Statistical analysis

The Kaplan–Meier curves were used to illustrate survival between treatment groups, and statistical assessment was performed using the log-rank test. For other parameters, the D’Agostino and Pearson omnibus normality test was used to test for normality. Multiple comparisons were analyzed using one-way analysis of variance (ANOVA) with Sidak’s *post hoc* correction or the non-parametric Kruskal–Wallis test with Dunn’s *post hoc* correction. Data are presented as mean + standard deviation (SD). A p-value of <0.05 indicated statistical significance. All statistical analyses and data visualizations were performed using GraphPad Prism v.9.0 (GraphPad Software, La Jolla, CA, USA).

## 3 Results

### 3.1 Levels of AAT are higher during acute sepsis in mice PL fluid but not in plasma

**Figure 1:**
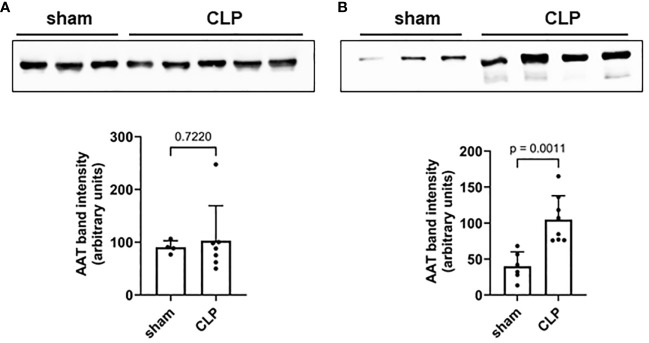
Mouse plasma **(A)** and PL fluid **(B)** levels of AAT. Levels of endogenous AAT in plasma **(A)** and peritoneal lavage **(B)** were semi-quantified in sham (n = 6) and septic (n = 8) mice 20 h after induction of high-grade sepsis. Representative Western blots for AAT and semi-quantitative densitometric analyses are shown. PL, peritoneal lavage; AAT, alpha1-antitrypsin.

Mouse plasma and PL fluid samples were collected 20 h after high-grade sepsis induced by CLP surgery. No differences in AAT plasma levels were observed between sham and septic mice (**Figure 1A**). However, significantly higher AAT levels were observed in PL fluid in septic mice compared to sham mice (**Figure 1B**).

### 3.2 Treatment with AAT improves survival and reduces the systemic inflammatory response of septic mice

**Figure 2:**
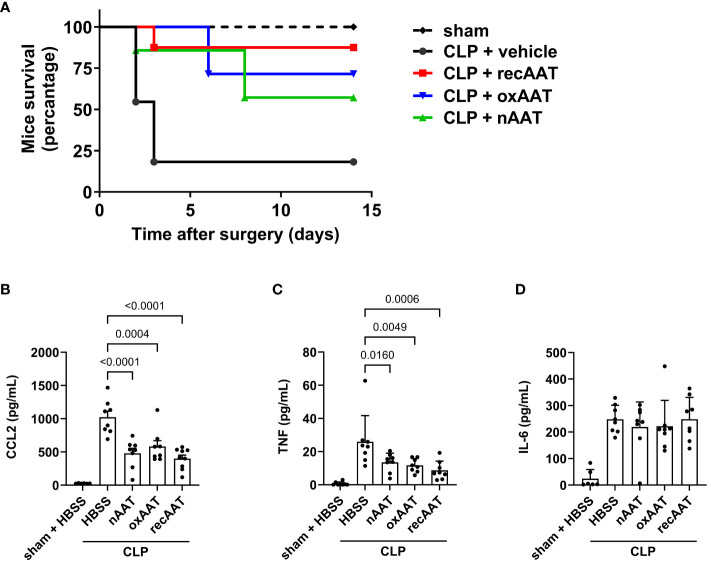
Effects of AAT preparations on survival and systemic inflammatory response in polymicrobial sepsis model. **(A)** Mid-grade sepsis was induced in mice by CLP surgery with ligation of 50% of the cecum length (n = 8 mice per group). Survival was monitored daily for 14 days, Kaplan–Meier curves were generated, and Gehan–Breslow–Wilcoxon test was performed for statistical analysis. Sham-operated mice served as controls (n = 6 mice per group). Plasma levels of inflammatory markers were determined at 24 h after sham or CLP surgery: CCL2 **(B)**, TNF **(C)**, and IL-6 **(D)**. Data are presented as the mean (SD), and p < 0.05 was considered significant. AAT, alpha1-antitrypsin; CLP, cecal ligation and puncture.

In survival experiments, mid-grade sepsis was induced in mice by CLP surgery (n = 8/group) with ligation of 50% of the cecum length. This semi-lethal CLP mouse model with a survival rate of 40% is the most suitable CLP model to investigate the protective effects of therapeutic interventions on sepsis-related mortality (21). Sham-operated mice (n = 6) served as controls. Septic mice received 200 mg/kg recAAT, human nAAT, or oxAAT or were injected with vehicle (HBSS buffer) immediately after CLP surgery. As shown in **Figure 2A**, only 18% of vehicle-treated mice survived, compared to 87%, 71%, and 57% in the groups treated with recombinant, oxidized, and plasma-derived native AAT, respectively. Mice treated with all three forms of AAT showed significantly better outcomes compared to HBSS treatment (recAAT p < 0.01, nAAT p < 0.05, and oxAAT p < 0.01 *vs.* vehicle treatment).

The systemic inflammatory response was assessed by measuring the plasma concentration of the pro-inflammatory cytokines TNF and IL-6 and the chemokine CCL2. As expected, CLP-induced peritonitis was associated with strong systemic upregulation of all three pro-inflammatory mediators. Treatment with any of the three forms of AAT significantly reduced plasma levels of CCL2 and TNF but had no effect on IL-6 levels (**Figures 2B–D**, respectively).

### 3.3 Therapy with AAT has no effect on the bacterial load in peritoneal fluid, blood, and spleen

**Figure 3:**
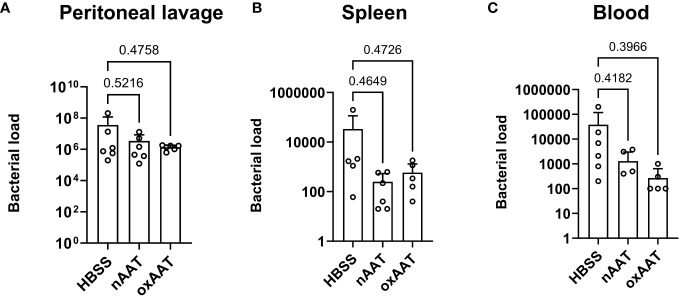
Effect of treatment with nAAT and oxAAT on bacterial load in polymicrobial sepsis model. High-grad sepsis was induced in mice by CLP surgery (n = 6 mice per group). Bacterial load analysis was performed in peritoneal lavage (PL) fluid **(A)**, spleen **(B)**, and blood **(C)** samples obtained at 20 h after surgery. Data are presented as the mean (SD). Negative controls with samples of non-CLP-treated mice remained sterile (data not shown). nAAT, plasma-derived native; oxAAT, oxidized nAAT; CLP, cecal ligation and puncture.

To investigate possible mechanisms underlying the beneficial effects of AAT on survival and the systemic inflammatory response, high-grade sepsis was induced by CLP surgery with ligation of 75% of the cecum length. The *in vivo* clearance of bacteria was examined in the PL fluid, blood, and spleen 20 h post-CLP operation. Colony-forming bacterial loads in blood, PL fluid, and spleen homogenates were slightly lower, but not significantly changed, in the septic mice treated with human nAAT or oxAAT compared to the vehicle-treated septic mice. These results were observed using Columbia blood agar plates (**Figure 3**) as well as using Schaedler agar and MacConkey agar plates (data not shown).

### 3.4 Treatment with AAT reduces plasma markers of multi-organ failure in high-grade septic mice

**Figure 4:**
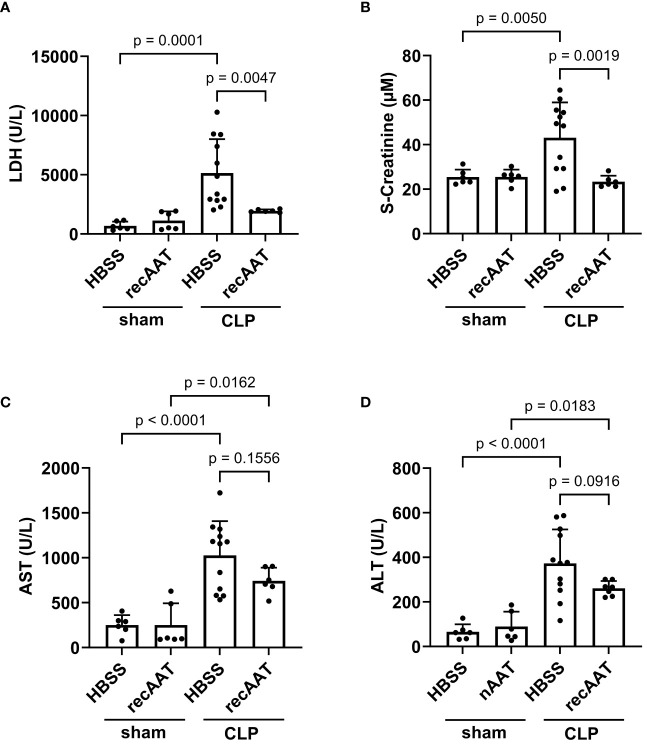
Effect of recAAT on multi-organ failure in polymicrobial high-grade sepsis mouse model. High-grad sepsis was induced in mice by CLP surgery (n = 12 and 9 mice per group for untreated and recAAT-treated mice, respectively). Sham-operated mice served as controls (n = 6 mice per group). Plasma analysis was performed at 20 h after sham or CLP surgery: **(A)** plasma lactate dehydrogenase (LDH) levels, **(B)** plasma creatinine levels, and **(C, D)** aspartate transaminase (s-AST) and alanine aminotransferase (s-ALT) levels, respectively. Data are presented as the mean (SD), and p < 0.05 was considered significant. recAAT, recombinant alpha1-antitrypsin; CLP, cecal ligation and puncture.

Since recAAT showed the best effect on mouse survival, the following short-term experiments (follow-up 20 h) were performed using recAAT only. High-grade sepsis was induced in mice by CLP surgery with ligation of 75% of the cecum length (n = 10–12 mice per group). Sham-operated mice served as controls (n = 6–8 mice per group). Plasma levels of lactate dehydrogenase (LDH) reflecting the degree of sepsis-induced overall tissue damage were determined in the plasma of sham- and CLP-operated mice 20 h after surgery. As expected, induction of sepsis resulted in a significant increase in LDH levels compared to sham mice. Plasma LDH levels in septic mice treated with recAAT were significantly lower compared to those in vehicle-treated septic mice and were not significantly different from those in sham mice (**Figure 4A**).

Next, renal function was estimated by measuring plasma creatinine after sham or CLP surgery. As shown in **Figure 4B**, plasma creatinine levels were significantly higher in septic mice treated with a vehicle, whereas creatinine levels in septic mice treated with recAAT did not differ from sham controls. Finally, we determined plasma levels of alanine aminotransferase (ALT) and aspartate transaminase (AST) as markers of sepsis-induced hepatocellular injury. ALT and AST levels were significantly higher in the plasma of vehicle-treated septic mice compared to sham animals (**Figures 4C, D**). Compared to vehicle-treated septic mice, the increase in AST and ALT levels was less pronounced in recAAT-treated septic mice, but without statistical significance.

### 3.5 Treatment with recAAT reduces the systemic and local inflammatory response in high-grade sepsis

**Figure 5:**
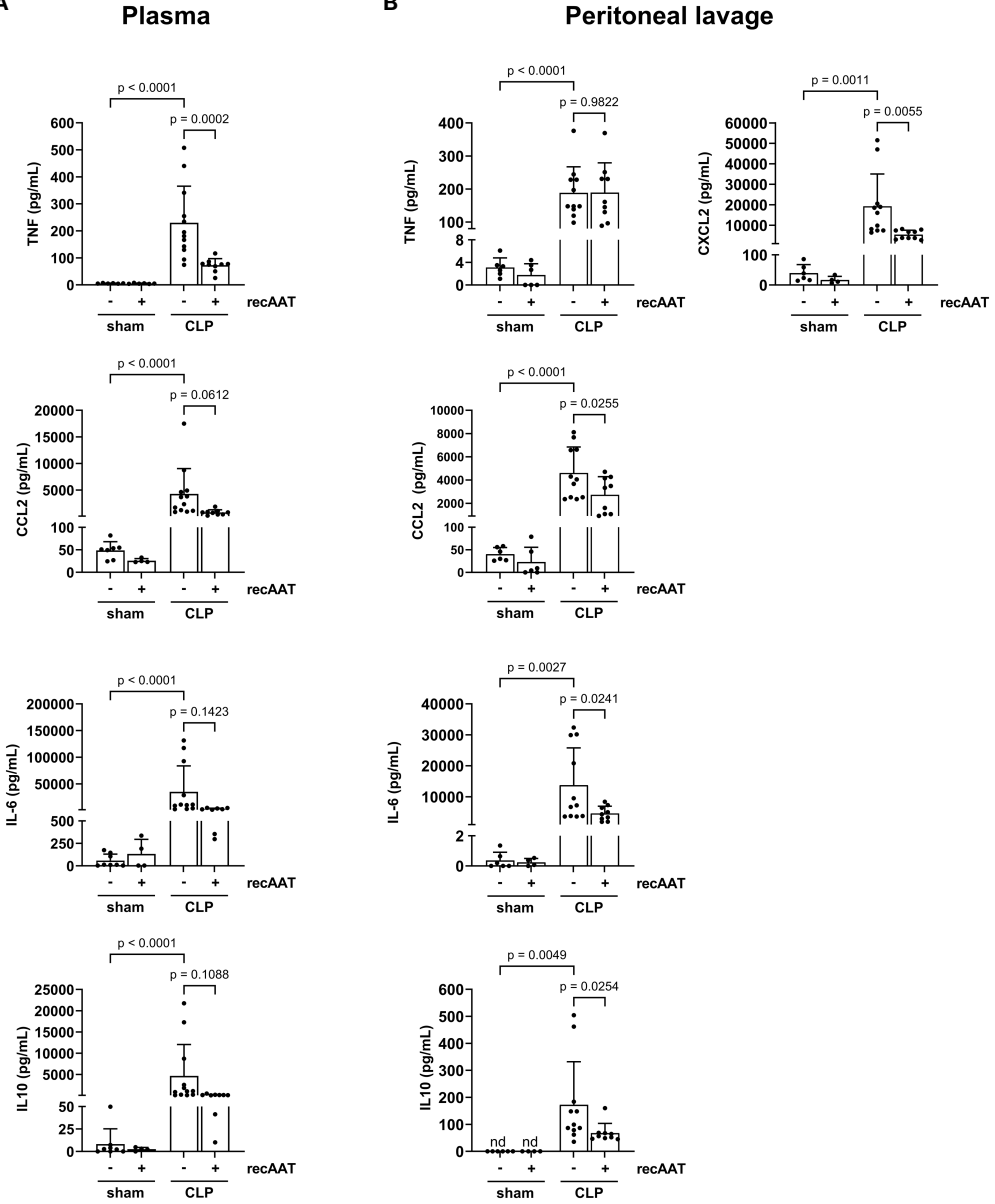
Effect of recAAT on CLP-induced systemic and local inflammatory response. High-grade sepsis was induced in mice by CLP surgery (n = 12 and 9 mice per group for untreated and recAAT-treated mice, respectively). Sham-operated mice served as controls (n = 6 mice per group). Blood **(A)** and peritoneal lavage fluid **(B)** sampling and measurements of pro-inflammatory mediators IL-6, CCL2, TNF, and anti-inflammatory IL-10 were performed at 20 h after surgery. Additionally, CXCL2 was measured in the peritoneal lavage fluid samples. Data are presented as the mean (SD), and p < 0.05 was considered significant. recAAT, recombinant alpha1-antitrypsin; CLP, cecal ligation and puncture.

The levels of inflammatory mediators were analyzed in plasma and PL fluid 20 h after CLP or sham surgery. As expected, CLP-induced peritonitis was associated with strong systemic (**Figure 5A**) and local (**Figure 5B**) upregulation of pro-inflammatory cytokines IL-6, CCL2, and TNF as well as upregulation of anti-inflammatory IL-10 compared to sham animals. Levels of neutrophil chemoattractant CXCL2 were also increased in PL of septic mice (**Figure 5B**). Compared to vehicle-treated septic mice, recAAT-treated mice showed significantly lower plasma TNF levels and a strong trend toward lower CCL2, IL-6, and IL-10 levels (**Figure 5A**). In contrast, in PL fluid, the levels of IL-6, CCL2, CXCL2, and IL-10 were significantly lower in septic mice treated with recAAT, whereas TNF levels were not affected (**Figure 5B**).

**Figure 6:**
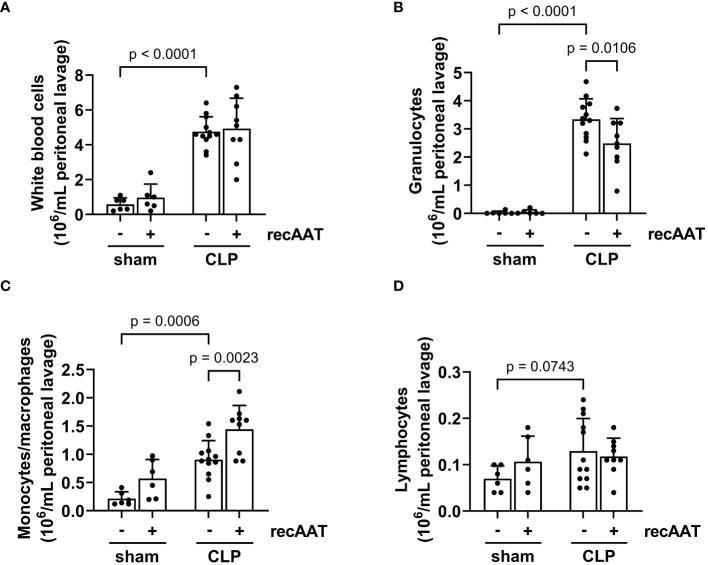
Effect of recAAT on CLP-induced leukocyte infiltration. High-grade sepsis was induced in mice by CLP surgery (n = 12 and 9 mice per group for untreated and recAAT-treated mice, respectively). Sham-operated mice served as controls (n = 6 mice per group). Peritoneal lavage was performed at 20 h after surgery, and flow cytometry (FACS) analysis of inflammatory cell populations, namely white blood ceels **(A)**, granulocytes **(B)**, monocytes and macrophages **(C)**, and lymphocytes **(D)**, was performed. Data are presented as the mean (SD), and p < 0.05 was considered significant. recAAT, recombinant alpha1-antitrypsin; CLP, cecal ligation and puncture; FACS, fluorescence-activated cell sorting.

Recruitment of inflammatory leukocytes such as neutrophils and monocytes into the abdominal cavity is a hallmark of abdominal sepsis (24). Therefore, we analyzed the total white blood cell counts and quantified the number of lymphocytes, monocytes, and granulocytes in the PL fluid 20 h after surgery. The total number of inflammatory cells in the PL fluid was greatly increased in septic mice compared to sham controls (**Figure 6A**), mainly due to an increase in granulocytes and monocytes (**Figures 6B, C**). The total number of lymphocytes did not change (**Figure 6D**). Treatment of septic mice with recAAT reduced granulocyte numbers (**Figure 6B**) but increased monocyte/macrophage content in the peritoneal cavity (**Figure 6C**).

### 3.6 Treatment with recAAT reduces capillary leakage and labile heme accumulation in the peritoneal cavity

**Figure 7:**
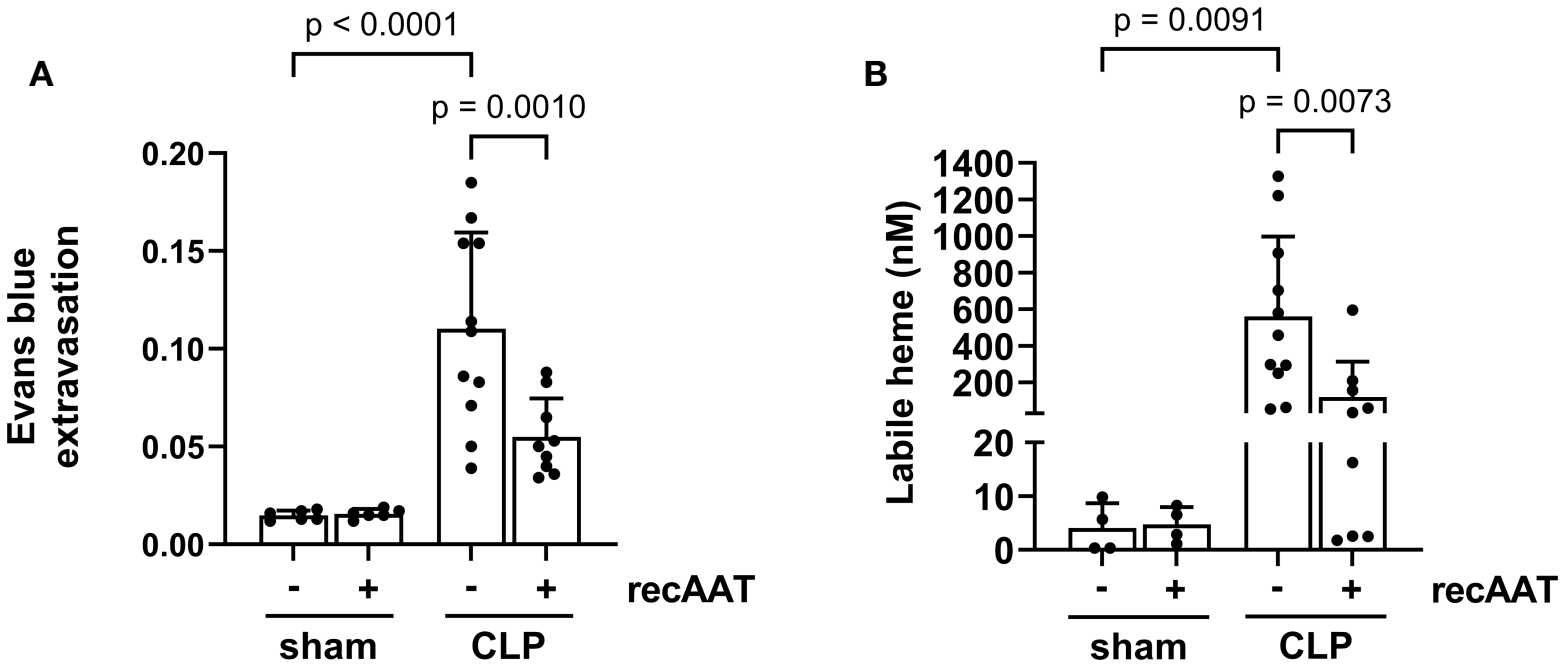
Effect of recAAT on endotoxemia-induced vascular leakage and intraperitoneal labile heme accumulation. High-grade sepsis was induced in mice by CLP surgery (n = 11 and 9 mice per group for untreated and recAAT-treated mice, respectively). Sham-operated mice served as controls (n = 6 mice per group). **(A)** After surgery, mice were i.v. injected with 0.25% w/v Evans blue and i.p. injected with vehicle or AAT. As a measure of capillary leakage, the ratio of Evans blue in the peritoneal lavage (PL) and the plasma after 20 h is displayed. **(B)** The concentration of labile heme was measured in PL obtained after 20 h from n = 9–11 mice and n = 4 per group for septic and control (sham) groups, respectively. Data are presented as the mean (SD); p-value < 0.05 was considered significant. AAT, alpha1-antitrypsin; recAAT, recombinant alpha1-antitrypsin; CLP, cecal ligation and puncture.

Vascular leakage caused by endotoxemia is a devastating feature of the disproportionate host immune response (25). Compared to sham surgery, a significant increase in vascular leakage was observed 20 h after the CLP procedure, as reflected by increased extravasation of Evans blue dye into the abdominal cavity. This sepsis-induced vascular hyperpermeability was significantly reduced by treatment with recAAT (**Figure 7A**).

Both vascular leakage and cell damage caused by endotoxemia can contribute to the local accumulation of free heme in the abdominal cavity (8). Indeed, we observed significantly higher levels of free heme in the PL fluid of septic mice compared to sham controls. Treatment with recAAT significantly reduced the amount of labile heme in the PL fluid of septic mice (**Figure 7B**).

### 3.7 RecAAT lowers lipopolysaccharide-induced release of pro-inflammatory mediators from mesothelial epithelium and primary peritoneal macrophages *in vitro*

**Figure 8:**
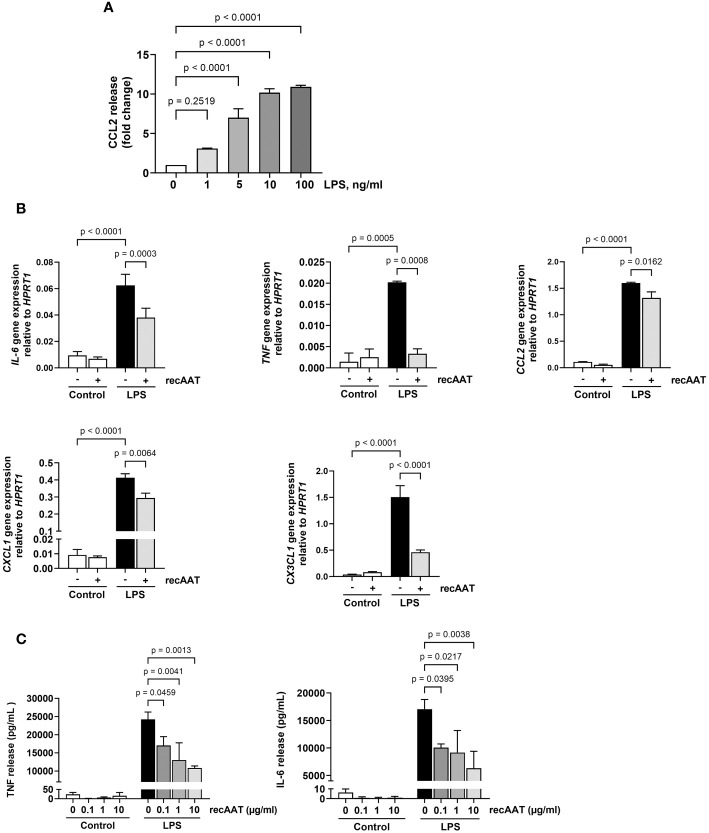
Effects of recAAT on LPS-induced release of pro-inflammatory mediators from immortalized mouse peritoneal mesothelial cells (MPMC) and primary peritoneal macrophages. **(A)** MPMC were stimulated with increasing concentrations of LPS for 24h CCL2 level in conditioned medium was assessed by ELISA. **(B)** MPMC were pre-incubated or not for 1 h with 1 mg/mL recAAT and then stimulated with 100 ng/mL LPS. Inflammatory mediator gene mRNA expression was assessed after 4 h. **(C)** Primary peritoneal macrophages were pre-incubated or not for 1 h with increasing concentrations of recAAT and then stimulated with 10 ng/mL LPS for 5 h. Unstimulated cells served as controls. The release of pro-inflammatory mediators was assessed in conditioned medium. Data are presented as the mean (SD) from four independent experiments; a p-value <0.05 was considered significant. recAAT, recombinant alpha1-antitrypsin; LPS, lipopolysaccharide.

Mesothelial epithelium covering the internal body cavities and organs, and resident peritoneal macrophages pose the first line of defense in abdominal bacterial sepsis. In the early stages of infection, bacterial endotoxins activate Toll-like receptors on the surface of these cells, leading to a fulminant release of pro-inflammatory and chemotactic mediators, representing a crucial event in the pathogenesis of sepsis (26). To test whether AAT impairs the LPS-induced release of pro-inflammatory mediators, immortalized MPMC and primary naïve MPMΦ were used for *in vitro* experiments. As shown in **Figure 8A**, exposure of MPMC to increasing concentrations of LPS ranging from 1 to 100 ng/mL for 24 h resulted in an LPS dose-dependent increased release of CCL2. In the next series of experiments, MPMC were stimulated with a constant dose of LPS (100 ng/mL) for 4 h and analyzed for the expression of the *IL-6*, *TNF*, *CCL2*, *CXCL1*, and *CX3CL1* genes. As expected, LPS upregulated the expression of all genes analyzed (**Figure 8B**). This LPS-induced inflammatory gene expression was significantly reduced in the presence of recAAT, whereas the expression of *TNF* was completely abolished (**Figure 8B**).

Similar results were obtained for primary peritoneal macrophages. recAAT reduced the cellular release of IL-6 and TNF in response to LPS in a concentration-dependent manner compared to LPS treatment alone (**Figure 8C**).

### 3.8 AAT prevents the enhancement of LPS-induced response by free heme in mouse primary MPMΦ and in immortalized mouse peritoneal mesothelial cells *in vitro*

**Figure 9:**
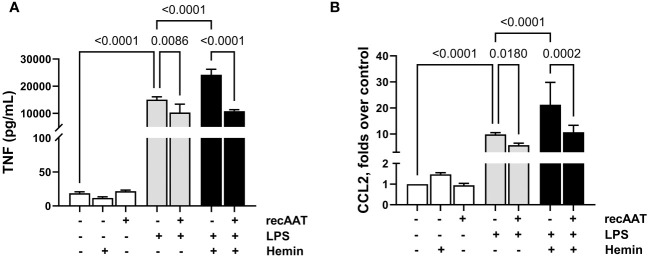
Effect of recAAT on LPS and free heme-induced TNF release from MPMΦ and CCL2 release from immortalized MPMC. Primary MPMΦ **(A)** and MPMC **(B)** were pre-incubated with 100 µg/mL recAAT for 1 h and then stimulated with 5 ng/mL LPS alone or in combination with 0.5 µM hemin for 24 h. The levels of TNF **(A)** and CCL2 **(B)** were measured in conditioned medium by CBA assay. Data are presented as the mean (SD) from three independent experiments performed in duplicates; p < 0.05 was considered significant. recAAT, recombinant alpha1-antitrypsin; LPS, lipopolysaccharide; MPMΦ, peritoneal macrophages; MPMC, mouse peritoneal mesothelial cells.

Recently, a synergistic effect of free heme on LPS-induced cytokine secretion in murine macrophages was demonstrated (27). Since we observed the accumulation of free heme in PL fluid from septic mice, in the following experiments, MPMΦ were stimulated with 5 ng/mL LPS alone or in the presence of 5 µM heme. While heme alone had no effect on TNF release into a conditioned medium, LPS-induced TNF release was significantly higher in the presence of heme. Notably, the addition of recAAT (100 µg/mL) to MPMΦ not only lowered LPS-induced TNF release but also completely blocked the synergistic effect of heme (**Figure 9A**). Similar results were obtained for MPMC stimulated with 5 ng/mL LPS alone or in the presence of 0.5 μM free heme. While free heme alone did not increase CCL2 levels in a conditioned medium, LPS-induced CCL2 release was significantly increased in the presence of heme (**Figure 9B**). Treatment of MPMC with recAAT not only reduced CCL2 release after LPS stimulation but also completely blocked the synergistic effect of free heme (**Figure 9B**).

### 3.9 AAT prevents free heme-induced cytotoxicity in immortalized mouse peritoneal mesothelial cells

**Figure 10:**
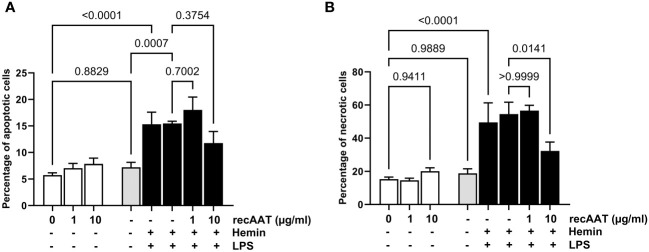
AAT reduces free heme-induced cytotoxicity in MPMC. MPMC were stimulated for 24 h with 5 ng/mL LPS, with 10 µM hemin separately or in combination in the presence of increasing concentrations of recAAT. The percentage of apoptotic **(A)** and necrotic **(B)** cells was analyzed by flow cytometry using apoptosis detection kit. Data are presented as the mean (SD) from four independent experiments performed in duplicates; p-value <0.05 was considered significant.

Cells were stimulated with 5 ng/mL LPS, 10 μM free heme, or a combination of both. LPS alone did not increase the percentage of apoptotic (AV+/PI−) or necrotic (AV+/PI+) cells. In contrast, cytotoxicity was observed after 24 h of cell culture with 10 μM heme and with the LPS/heme combination. The addition of recAAT (10 μg/mL) to the cell culture medium slightly but not significantly reduced the percentage of apoptotic cells, whereas it reduced the percentage of necrotic cells significantly (**Figure 10**).

## 4 Discussion

Various biomarkers are increased or decreased during sepsis, although the significance and exact biochemical function of many of them remain unclear. Acute phase proteins are involved in the host defense response and the regulation of inflammatory processes during sepsis, while AAT is one that increases significantly (three- to fourfold) within hours of inflammation or infection (28). AAT affects the course of inflammatory reactions by inhibiting neutrophil elastase and other proteases, interacting with various pro-inflammatory molecules, and exerting immunomodulatory effects, some of which are independent of anti-protease activity. Notably, however, insufficient plasma concentrations of AAT were observed in patients with severe sepsis or multi-organ failure. For example, delayed early increases in AAT and other acute phase proteins were found in non-survivors with sepsis (29). In addition, susceptibility to septic complications has been shown to be higher in lung transplant patients with congenital AAT deficiency than in patients with a normal genetic variant of AAT. Sepsis remained a leading cause of death in patients with AAT deficiency, even more than 6 months after lung transplantation, according to a Toronto study (30). Clinical studies typically describe plasma changes in AAT levels in septic patients, whereas no data are available on local AAT levels at the original site of inflammation.

In the current study, we used a well-established CLP model that resulted in polymicrobial peritonitis, translocation of bacteria into the blood (bacteremia), local and systemic inflammation, multiple organ dysfunction, and ultimately death (31–33). Using the CLP mouse model of high-grade sepsis with ligation of 75% of the length of the cecum, we observed no change in plasma AAT levels 20 h after surgery, while AAT levels in PL fluid were significantly higher compared to those in sham controls. These initial results from septic mice, together with previously published clinical data, suggest that adequate AAT levels in sepsis may be an important factor in controlling rapid and widespread inflammatory responses. Therefore, the main aim of the present study was to investigate whether the administration of exogenous AAT directly into the peritoneal cavity of septic mice can help control inflammation and multi-organ failure.

First, we used a half-lethal CLP mouse model of midgrade sepsis with ligation of 50% of the cecum length (21) to examine the effects of AAT on survival. Based on 14-day monitoring of mice, we clearly found that immediate i.p. injection of human nAAT, oxAAT, or recAAT significantly reduces mortality in CLP-septic mice compared with mice not receiving AAT. Consistent with the increased survival, septic mice treated with AAT showed significantly reduced systemic inflammation compared to untreated septic controls, as reflected by lower plasma levels of CCL2 and TNF. It is important to note that the oxidized form of nAAT, which lacks anti-protease activity, reduced mouse mortality in the same way as inhibitory active nAAT or recAAT proteins, suggesting that the observed protective effect of AAT is not entirely due to the anti-protease activity. Previous mouse studies based on intratracheal quartz installation or cigarette smoke exposure reported that oxAAT does not inhibit elastase but, like nAAT, suppresses neutrophil influx and expression of inflammatory mediators (34, 35). In a mouse model of pneumonia, we found that AAT without anti-protease activity retained its potent anti-inflammatory and immunomodulatory effects (36, 37). Oxidized forms of AAT are estimated to be present in human inflammatory exudates in an amount of approximately 5%–10% of total AAT (38). However, characterizing the biochemical properties and biological functions of oxAAT requires separate *in vitro* and *in vivo* approaches, which was beyond the scope of this study. Therefore, all our further *in vivo* and *in vitro* experiments were performed using inhibitory active nAAT or recAAT proteins.

The positive effect of augmentation with AAT on the survival of septic mice prompted us to conduct a second series of experiments focused on the putative biological effects of AAT. Here, CLP surgery with ligation of 75% of the cecum length was used to produce high-grade sepsis. Plasma and PL fluid were collected at 20 h after surgery for further analysis. First, consistent with the survival data, we found that LDH levels, reflecting the degree of overall damage caused by sepsis, as well as s-creatinine, a marker of renal function, were lower in AAT-treated compared to non-treated septic mice. Although without statistical significance, plasma markers of hepatocellular damage such as s-ALT and s-AST were also reduced. These results demonstrated a broad protective potency of AAT even in severe high-grade sepsis.

Bacterial burden, exaggerated inflammatory response, increased vascular leakage, accumulation of free heme released during hemolysis, and death of cells with high hemoprotein content are critical processes in the pathogenesis of severe sepsis (8, 9, 31–33). Therefore, in the following experiments, we investigated the putative biological effects of AAT on these processes.

Clinical and animal model-based studies reported that AAT can reduce bacterial colonization and bacterial burden (13, 39–42). These latter findings led to speculation that AAT may have a direct impact on bacterial growth, thereby protecting septic mice from developing severe organ damage and death. In our experiments, AAT had no significant impact on the number of bacteria in blood, peritoneal lavage fluid, or spleen samples. Hence, the reduction of the bacterial load does not appear to be the mechanism behind the effect of AAT on reduced mouse mortality.

The effect of AAT on systemic and local inflammatory responses was further investigated by the measurement of inflammatory mediators in plasma and PL fluid. We found that AAT-treated septic mice have significantly lower PL fluid levels of IL-6, CCL2, CXCL2, and IL-10, but not TNF, while plasma levels of TNF and CCL2, but not IL-6, were significantly lower compared to those in untreated septic controls. Thus, AAT appears to modulate IL-6, TNF, and IL-10 levels in mouse plasma and PL fluid differently. This may be related to the mode of administration of AAT, but more complex mechanisms cannot be excluded.

The recruitment of phagocytic cells, namely, neutrophils and monocytes, to the peritoneal cavity depends critically on the upregulation of adhesion molecules on the endothelium by IL-6 and TNF and the levels of the chemokines CXCL2 and CCL2. Although neutrophils kill invading bacteria, strong infiltration and/or delayed apoptosis of neutrophils may have deleterious effects in sepsis (43–45). CXCL2 and neutrophil influx into the peritoneal cavity of septic mice were significantly reduced by AAT treatment. These results are consistent with other reports showing that the anti-inflammatory effect of AAT is related to the inhibition of neutrophil infiltration and neutrophil-mediated tissue damage (45). Surprisingly, despite the decreased level of CCL2 chemokine, monocyte/macrophage infiltration was significantly increased by AAT treatment (**Figure 6C**).

We previously reported that AAT initially facilitates acute endothelial responses to TNF, followed by selective inhibition of TNF-induced self-amplification, which may help resolve inflammation (46). Since TNF affects monocyte infiltration and functions (47), high TNF levels may be related to increased numbers of monocytes/macrophages in the PL fluid of AAT-treated mice. Both peripheral macrophages and monocyte-derived macrophages transcribe the *serpina1* gene and secrete AAT protein, which affects the anti-inflammatory functions of macrophages (48, 49). Therefore, increased numbers of monocytes/macrophages in the PL fluid may contribute to local AAT levels.

In recent decades, researchers have shown that high levels of free (labile) heme reflect the pathogenesis of severe sepsis regardless of pathogen load (8, 50). High heme levels were reported in patients with sepsis, and similar findings have been observed in experimental models of CLP murine sepsis (8, 51). For example, free heme exerted cytotoxicity *in vitro* and exacerbated tissue injury in a rat model of polymicrobial sepsis (9). However, the data about free heme accumulation directly at the site of initial infection were not available up to now. In this study, we did not measure haptoglobin, bilirubin, or other parameters of hemolysis in the plasma samples. However, we observed a strong accumulation of free heme in the peritoneal cavity of septic mice. The source of free heme in the peritoneal cavity may be both extravasation of free heme generated by sepsis-induced hemolysis from the circulation and also its release from hepatocytes that underwent sepsis-induced necrosis and apoptosis. Hepatocytes contain large amounts of heme incorporated into microsomal hemoproteins, such as cytochrome P450 (CYP450) (52).

In blood plasma, heme is scavenged by hemopexin and also by albumin, α1-microglobulin, and α1-antitrypsin (53). Severe sepsis is associated with reduced plasma concentrations of hemopexin, the top heme-binding protein (51). Albumin is a low-affinity but high-capacity heme scavenger that attenuates heme-mediated vasoconstriction *in vivo* and prevents heme-mediated cytotoxicity *in vitro* (9). However, plasma albumin levels in severe septic patients also decrease when compared to those in non-septic patients (54). We and other researchers have shown that AAT, similarly to albumin, binds free heme and neutralizes its cytotoxic effects (11, 55, 56). In this study, we demonstrate that a strong accumulation of free heme in the peritoneal cavity of septic mice was significantly reduced by AAT treatment.

Free heme can promote and exacerbate inflammation through various mechanisms. For example, in endothelial cells, heme-induced upregulation of the adhesion molecules E-selectin, P-selectin, intercellular adhesion molecule 1 (ICAM-1), and vascular cell adhesion molecule 1 (VCAM-1) has been shown to promote leukocyte infiltration (57, 58). Heme can also act as an endogenous agonist of TLR2/4 receptors (59, 60) and directly induce the secretion of pro-inflammatory mediators such as the neutrophil chemoattractant CXCL2 (61). There is abundant evidence that infectious sepsis in both humans and mice with polymicrobial sepsis results in robust activation of complement. Major complement activation products such as C3a/C5a anaphylatoxins and their receptors and the terminal complement activation product C5b-9 cause dysfunction of the innate immune system and contribute significantly to exaggerated early pro-inflammatory responses, followed by decline of the innate immune system, leading to immunosuppression and multi-organ dysfunction (62). Complement system activation by free heme has been shown *in vitro* and *in vivo* resulting in tissue deposits of complement C3 and C5b-9 primarily in the kidneys (63). In addition, free heme can directly induce endothelial permeability by affecting NF-κB signaling through activation of TLR4 and by inducing an acute signaling cascade through p38 MAPK and HSP27, leading to barrier dysfunction (64). Therefore, the reduced renal dysfunction, inflammation, and vascular leakage observed in septic mice treated with AAT may be related to the property of AAT to neutralize the free heme.

Resident peritoneal macrophages and mesothelial epithelial cells, which line the internal body cavities, form the first line of defense in abdominal bacterial sepsis. In the early stages of infection, bacterial endotoxins activate these cells, leading to a fulminant release of pro-inflammatory and chemotactic mediators, which is a crucial event in the pathogenesis of sepsis. Our data from *in vitro* experiments using MPMC and primary mouse peritoneal macrophages confirmed that AAT significantly lowers LPS-induced IL-6, TNF, CXCL1, and CCL2 production. Indeed, CCL2 and CXCL1 not only control chemotactic chemokine but also control the production of inflammatory cytokines, such as TNF and IL-6 (65). It is also important to note that LPS in synergy with labile heme is a much more potent cell activator than LPS alone (66). Our data confirm this assumption by showing that MPMΦ stimulated with LPS in the presence of heme release significantly higher amounts of TNF compared to cells stimulated with LPS alone. Similarly, MPMC released significantly higher amounts of CCL2 in response to LPS/heme combination compared to LPS alone. In these *in vitro* experiments, cell pretreatment with AAT not only significantly reduced the LPS effect but also completely blocked the synergistic effect of heme. In addition, AAT significantly reduced cell death in response to heme or LPS/heme combination. Interestingly, this reduction in cell death was not related to the reduction in apoptotic cells. This is in line with our previous observation that incubation of human neutrophils for 5 h with 4 mM hemin only slightly and not significantly increased the number of apoptotic cells but strongly increased the number of non-viable neutrophils (56). Some researchers suspect that heme induces cell ferroptosis via mitochondrial dysfunction (67). In addition, free heme is a well-known inducer of heme oxygenase-1 (HO-1), particularly in monocyte/macrophage cells (53), which has been implicated as a key mediator of inflammatory cell and tissue injury, as validated in preclinical models of acute lung injury and sepsis (68). In ferroptosis, HO-1 may play a pro-death role by enhancing iron release (69). The sequestration of free heme by AAT, leading to decreased HO-1 expression, may have an anti-ferroptosis effect and partially explain increased amounts of monocytes/macrophages in the peritoneal cavity of AAT-treated septic mice. Further studies on the anti-ferroptosis effect of AAT are currently underway.

The results of our study suggest that selective regulation of local immune cell populations and inflammatory cytokine/chemokine levels in the early stages of sepsis by AAT prevents hyperinflammation and death in septic mice. Some beneficial effects of AAT are related to its ability to scavenge free heme and prevent heme toxicity in synergy with bacterial endotoxins. An optimal AAT dose administered rapidly enough to patients with acute sepsis would allow additional time for more targeted interventions. Moreover, it can be hypothesized that the combined use of AAT, as a free heme scavenger, with an LPS neutralizer, such as alkaline phosphatase (70, 71), may be useful in treating sepsis and may be tested in clinical trials. Before AAT therapy is tested in clinical practice, important future research is needed to define how long after diagnosis of sepsis treatment with AAT can be considered anti-inflammatory and protective. This idea can be further tested in translational preclinical models and small patient cohorts.

Given the observed advantages of recAAT produced by CHO cells, this protein may be superior to AAT purified from human plasma because the recAAT preparation is homogeneous, has high quality and glycosylation consistency, and poses no risk of disease transmission.

## Data availability statement

The original contributions presented in the study are included in the article/**Supplementary Material**. Further inquiries can be directed to the corresponding author.

## Ethics statement

The animal study was approved by Lower Saxony Office for Consumer Protection and Food Safety, LAVES, no. 21-3761. The study was conducted in accordance with the local legislation and institutional requirements.

## Author contributions

JZ: Data curation, Investigation, Writing – review & editing, Formal analysis, Project administration. ST: Investigation, Writing – review & editing. SS: Investigation, Writing – review & editing. VV: Investigation, Writing – review & editing. AK: Validation, Writing – review & editing. RL: Investigation, Writing – review & editing. JH: Formal analysis, Writing – review & editing. SI: Validation, Writing – review & editing. FW: Methodology, Writing – review & editing. TW: Conceptualization, Writing – review & editing. HH: Conceptualization, Validation, Writing – review & editing. SJ: Conceptualization, Supervision, Writing – original draft. NS: Conceptualization, Data curation, Formal analysis, Investigation, Project administration, Supervision, Validation, Writing – original draft.
