# Supplementary material for: Alpha1-antitrypsin improves survival in murine abdominal sepsis model by decreasing inflammation and sequestration of free heme
Source: Front Immunol. 2024 Mar 18;15:1368040. doi: 10.3389/fimmu.2024.1368040 (PMC10982482; doi:10.3389/fimmu.2024.1368040)
Supplement: Supplementary file 1 [file DataSheet_1.docx]

**Supplements**

**SybrGreen Primers for qPCR**

Murine CCL2 FP: 5'-TTA AAA ACC TGG ATC GGA ACC AA-3' (Eurofins Genomics)

Murine CCL2 RP: 5'-GCA TTA GCT TCA GAT TTA CGG GT-3' (Eurofins Genomics)

Murine mIL-6 FP: 5'-TAG TCC TTC CTA CCC CAA TTT CC-3' (Eurofins Genomics)

Murine mIL-6 RP: 5'-TTG GTC CTT AGC CAC TCC TTC-3' (Eurofins Genomics)

Murine CXCL1 FP: 5'-CTG GGA TTC ACC TCA AGA ACA TC-3' (Eurofins Genomics)

Murine CXCL1 RP: 5'-CAG GGT CAA GGC AAG CCT C-3' (Eurofins Genomics)

Murine TNFα FP: 5'-CAG CCT CTT CTC ATT CCT GC-3' (Eurofins Genomics)

Murine TNFα RP: 5'-GGT CTG GGC CAT AGA ACT GA-3' (Eurofins Genomics)

Murine CX3CL1 FP: 5'-ACG AAA TGC GAA ATC ATG TGC-3' (Eurofins Genomics)

Murine CX3CL1 RP: 5'-CTG TGT CGT CTC CAG GAC AA-3' (Eurofins Genomics)
